# Intraoperative nociceptive and clinical comparisons between ventral midline and flank ovariectomy in feral and stray cats

**DOI:** 10.1177/1098612X241283626

**Published:** 2024-12-24

**Authors:** João Martins, Afonso Grossinho, Liege Martins, Patrícia Cabral, José Diogo dos-Santos, Sónia Campos

**Affiliations:** 1 Faculty of Veterinary Medicine of Lisbon, Lusófona University, Lisbon University Center, Portugal; 2 Animal and Veterinary Research Center (CECAV) Faculty of Veterinary Medicine Lusófona University-Lisbon University Centre; 3 Associate Laboratory for Animal and Veterinary Sciences (AL4AnimalS), Lisbon, Portugal

**Keywords:** Ovariectomy, flank, midline, nociception

## Abstract

**Objectives:**

The aim of the present study was to evaluate the differences in intraoperative nociception, incision size and operative time between midline (OVE*m*) and flank ovariectomy (OVE*f*) in feral or stray cats.

**Methods:**

Two groups of animals, the OVE*m* group (n = 19) and the OVE*f* group (n = 19), were evaluated at six intraoperative time points. Cats assigned to both groups were premedicated with dexmedetomidine (20 μg/kg IM) and methadone (0.2 mg/kg IM). General anaesthesia was induced with intravenous propofol to effect and maintained with isoflurane in 100% oxygen. The data collected included heart rate, non-invasive systolic and median blood pressure, respiratory rate, weight, body condition score, surgical time, incision size and rescue analgesia.

**Results:**

Significant differences were observed between surgical approaches, with larger incision size (*P* <0.001) and longer surgery time (*P* = 0.04) in the OVE*m* group. No significant differences between surgical approaches were detected regarding intraoperative nociception (*P* >0.05).

**Conclusions and relevance:**

Based on intraoperative nociception, there is no strong reason to advocate a preferred surgical approach for feline OVE in sterilisation programmes; however, considering surgery time and incision size, the OVE*f* approach may contribute to the sterilisation of more animals and reduce the risk of wound dehiscence as it allows for smaller incisions to be made in animals where postoperative wound control is difficult or impossible.

## Introduction

Trap-neuter–return programmes are an important part of community efforts to prevent reproduction and overpopulation in unsocialised feral cats and socialised stray cats.^1–3^ There is evidence that these programmes improve the quality of life of individual cats and colonies and reduce sheltering and euthanasia of unwanted and unowned cats.^2,4^ In addition to improving animal welfare, these programmes are important from a One Health perspective, as human interaction with feral cat populations can pose a public health risk, increasing the risk of zoonotic diseases, injuries from minor scratches to bites and more serious infections in humans.^4^

Although these programmes are high-volume sterilisation services, high-quality care should be provided to each animal to improve the quality of patient care, reduce risks and improve patient outcomes,^2^ with the application of best practices in surgery, anaesthesia and pain control being of paramount importance.

In female cats, ventral midline and flank approaches are acceptable for ovariohysterectomy and ovariectomy (OVE) in paediatric and adult cats.^2,5–8^ Research exists to determine the best approach, with some authors citing the surgical advantages of the flank approach over the midline approach, such as less bleeding, wound inflammation or infection,^3,9^ less risk of wound dehiscence and evisceration,^10,11^ and economic advantages,^9^ while others cite the surgical disadvantages of the flank approach, such as limited visualisation of the abdominal cavity in the event of complications.^9,10^ Research on these techniques is difficult to compare owing to differences between studies,^12^ and research on nociception/pain associated with both procedures is scarce or non-existent. One study found differences in postoperative pain between the two approaches, but these were insignificant.^12^ The authors suggested that there may be differences between the sensitivity of the flank and midline skin or the musculature of the linea alba and flank following surgical trauma, or that less visceral pain may be associated with the improved visibility and accessibility of the uterus provided by the midline approach.

Although limited published data evaluate perioperative pain using both approaches,^8,11^ the authors are unaware of any studies comparing intraoperative nociception in cats associated with midline ovariectomy (OVE*m*) and flank ovariectomy (OVE*f*).

Intraoperative nerve damage^13^ and surgical trauma are important causes of chronic (or persistent) postoperative pain (CPOP). In humans, data suggest that a large number of patients develop CPOP after routine surgery^14^ and that CPOP can also be identified as a postoperative complication in veterinary medicine.^13^

Considering the difficulty in assessing postoperative pain in feral or stray cats and the possible impact that intraoperative nociception due to abdominal organ handling may have on increased postoperative pain and the development of CPOP,^7^ the authors propose to investigate whether there are differences in intraoperative nociception between OVE*m* and OVE*f*. We hypothesised that OVE*m* would be associated with less intraoperative nociception when compared with OVE*f*.

## Materials and methods

### Study population

All cats were feral or stray domestic shorthair adult queens enrolled in a sterilisation programme. Only cats classified as category I by the American Society of Anesthesiologists (ASA) were included in the study. Cages were weighed before the cats were trapped and transported to know the weight of the cats before drug administration. Paediatric and pregnant cats, or those with anomalies on physical examination, serum biochemistry or haematology, were excluded. Written informed consent was obtained from the responsible individuals for all the animals enrolled.

Ethical approval was obtained from the Comissão de Ética e Bem-Estar Animal at the Faculty of Veterinary Medicine of Lusófona University (11-2023).

An a priori power calculation was performed using the G*Power 3.1.9.7 statistical package (Heinrich Heine Universität Düsseldorf), indicating that 38 cats were needed to achieve 90% power and a 0.4 size effect, with a significance criterion of α = 0.05 error level, to detect clinically relevant differences in heart rate or systolic and mean arterial pressure. This study was randomised using a random sequence generator (https://www.random.org). At randomisation, 19 cats were allocated to the OVE*m* group and 19 cats to the OVE*f* group.

Cats were trapped the day before surgery and were admitted to the hospital the next day in individual cages and placed in a quiet room. Food, but not water, was withheld overnight before surgery. Cats were discharged on the day of surgery and released the day after surgery.

### Anaesthetic protocol

Cats allocated to the OVE*m* and OVE*f* groups followed a standardised anaesthetic protocol. Preanaesthetic medication included an intramuscular (IM) injection of dexmedetomidine hydrochloride (20 μg/kg, Dexdomitor 0.5 mg/ml; Orion) and methadone hydrochloride (0.2 mg/kg, Semfortan 10 mg/ml; Dechra). Cats were allowed to rest undisturbed in their cages until a sufficient level of sedation was achieved. Physical examination and blood sampling from the jugular vein for serum biochemistry and haematology were performed. The hair over the cephalic vein, the ventral abdomen (OVE*m* group) or the left flank (OVE*f* group) was clipped. An intravenous (IV) catheter was placed in a cephalic vein. After premedication, IV fluid therapy (3–5 ml/kg/h) with lactated Ringer’s solution (Lactated RingerVet; B Braun Medical) was administered until the end of surgery.

Preoxygenation for 3 mins was provided using a face mask. General anaesthesia was induced 15 mins after premedication with IV propofol (Propofol 10 mg/ml; B Braun Medical) administered to effect. After application of 0.1 ml of 2% lidocaine (Lidocaine 20 mg/ml; B Braun Medical) to the larynx, the trachea of the cats was intubated with a 3.5–4.5 mm cuffed endotracheal tube. General anaesthesia was maintained by spontaneous breathing of isoflurane (IsoFlo; Zoetis) delivered in oxygen via an Ayres T-piece with a Jackson-Rees modified non-rebreathing system (fraction of inspired oxygen 100%, fresh gas flow 250 ml/kg). The mean target-inspired fraction of isoflurane (F_i_ISO) concentration was set at 1% ± 0.1% for the intraoperative period to maintain the surgical plane of anaesthesia. After induction of general anaesthesia, the eyes were lubricated (Lubrithal Eye Gel; Dechra). Active warming was provided by a heating blanket (Thermal Blanket Carbonvet cage; B Braun Medical).

During anaesthesia, heart rate (HR; beats/min), respiratory rate (*f*_R_; breaths/minute), end-tidal carbon dioxide (CO2) (mmHg), inspiratory fraction of CO2 (mmHg), oesophageal temperature, oxygen haemoglobin saturation (%) and end-tidal isoflurane (%) were monitored. Blood pressure was measured using a non-invasive oscillometric method, with a number 2 cuff placed around the antebrachium of the right or left thoracic limb. Systolic arterial pressure (SAP) and mean arterial pressure (MAP) were measured at intervals of 3 mins. Parameters were monitored using an anaesthesia monitor (BeneVision N15; Mindray). Anaesthetic depth was assessed by monitoring trends in HR, *f*_R_, SAP, MAP, eye position, jaw tone and palpebral reflex.

When hypotension was detected (SAP <90 mmHg or MAP <60 mmHg), a 20% reduction in isoflurane and/or a 10 ml/kg bolus of lactated Ringer’s solution was administered; blood pressure was reassessed 5 mins later and the bolus repeated if necessary.

During surgery, rescue analgesia was administered if two or three variables (HR, MAP or *f*_R_) increased >20% from the preincision values. The rescue analgesia consisted of an IV bolus of fentanyl (2 μg/kg, Fentadon 50 µg/ml; Dechra).

HR, *f*_R_, oral mucous membrane colour and temperature were monitored at the end of general anaesthesia. Intravenous fluids were stopped and the cephalic vein catheter and endotracheal tube were removed. At the end of surgery, meloxicam (0.2 mg/kg, Metacam 2 mg/ml; Boehringer Ingelheim Vetmedica) was administered by subcutaneous injection for postoperative analgesia. Cats were allowed to recover in a clean individual cage.

### Surgical technique

Two senior surgeons were assigned to cases before disclosure of the OVE approach to reduce bias. The size of the skin incision in both OVE techniques was measured using a sterilised metal ruler in each case and was at the discretion of the surgeon assigned to the surgery to eliminate bias.

Cats in the OVE*m* group were placed in dorsal recumbency using a metal positioner, placed under the thermal blanket and a ventral midline laparotomy was performed caudal to the umbilicus.^8,15,16^ The uterus was identified by digital palpation, the ovary was exteriorised and OVE was performed with two ligatures using a 2 metric absorbable glyconate monofilament suture (Monosyn; B Braun Surgical) on the ovarian pedicle and the uterine horn near the proper ligament using the three-clamp technique on both structures.^8^ The ovary was removed after cutting between the haemostats and the procedure was repeated in the contralateral ovary.^16^ The ventral linea alba and the subcutaneous tissue were closed using the same absorbable glyconate monofilament (Monosyn; B Braun Surgical) in a simple continuous pattern. The skin was closed using the same absorbable glyconate monofilament in a simple continuous intradermal pattern.^16^

Cats assigned to the OVE*f* group were placed in right lateral recumbency with the pelvic limbs extended caudally.^8,15,16^ A skin incision was made after visualisation of an equilateral triangle with vertices at the greater trochanter, the wing of the ilium and the midpoint of the proposed incision.^8,15,16^ The abdominal cavity was entered by sequentially incising the skin, subcutaneous fat, external aponeurosis, internal and transverse abdominal oblique muscles, and peritoneum in a dorsal to ventral direction.^8,10,15,16^ The left uterine horn was identified, the respective ovary was exteriorised and OVE was performed with two ligatures using a 2 metric absorbable glyconate monofilament suture (Monosyn; B Braun Surgical) on the ovarian pedicle and the uterine horn near the proper ligament, using the three-clamp technique on both structures. The procedure was repeated in the contralateral ovary after identification of the contralateral uterine horn.^8^ The muscle layers and the subcutaneous tissue were closed using the same absorbable glyconate monofilament suture (Monosyn; B Braun Surgical) in a simple continuous pattern, and the skin was closed using the same absorbable glyconate monofilament suture in a simple continuous intradermal pattern.

### Data collection

Two senior anaesthetists were responsible for drug preparation, premedication, induction, orotracheal intubation and data collection. Data were collected on weight, body condition score (BCS), incision size and duration of surgery. Six surgical times were defined for both groups. Data on the outcome variables HR, *f*_R_, SAP and MAP were collected before skin forceps application (T0, baseline), at skin forceps application (T1), at skin incision (T2), at removal of the first ovary (T3) and the second ovary (T4), and finally at abdominal closure (T5). After application of the surgical stimuli (T1, T2, T3, T4 and T5), the data collected were the maximum values obtained for the haemodynamic variables (HR, SAP, MAP and *f*_R_). Data were collected before the surgeon proceeded to the next surgical stimulus. Rescue analgesia was recorded as ‘yes’ or ‘no’ during the intraoperative period and the surgical time during which the rescue analgesia was administered was also registered.

### Statistical analysis

Statistical analysis was performed using Jamovi version 2.5.3 computer software (https://www.jamovi.org). The normality of continuous numerical variables was assessed using the Shapiro–Wilk test. The results are presented as mean ± SD or median (range) for continuous variables and as frequencies for ordinal variables.

Intragroup repeated measures were analysed using the Kruskal–Wallis test followed by post-hoc pairwise comparisons. An independent *t*-test or Mann–Whitney U-test was used to compare the measured variables between the groups. Rescue analgesia was assessed using the χ^2^ test. The significance level was set at *P* <0.05.

## Results

A total of 38 ASA I, female domestic shorthair cats undergoing OVE were included in the study, with 19 cats assigned to the OVE*m* group and 19 cats assigned to the OVE*f* group. Age was unknown owing to their origin.

Cats in the OVE*m* and OVE*f* groups had a mean weight of 2.9 ± 0.5 kg and 2.9 ± 0.8 kg, respectively, and no significant differences were observed between the weights (*P* = 0.495).

In addition, no significant differences were observed between the median BCS in the OVE*m* and OVE*f* groups (*P* = 0.535).

During the intraoperative period, end-tidal CO2 and F_i_ISO were maintained in the range of 35–45 mmHg and 1% ± 0.1%, respectively.

Midline incisions (mean 2.6 ± 1.04 cm) were larger than flank incisions (mean 1.33 ± 0.5 cm) (*P* <0.001).

The surgery took longer in the OVE*m* group (mean 28.9 ± 11.6 mins) and significant differences were observed when compared with the OVE*f* group (mean 21 ± 5 mins) (*P* = 0.04).

In the OVE*m* group, no significant differences were observed between surgical times T0, T1, T2, T3, T4 and T5 (*P* >0.05) for haemodynamic variables HR, *f*R, SAP and MAP.

For the OVE*f* group, a significant difference was observed in the HR (*P* = 0.005) and SAP (*P* = 0.005) variables, with a significant increase in HR at T4 and a significant increase in SAP at T3 and T4. The haemodynamic variables *f*R and MAP did not show significant variations over the surgical time points (*P* >0.05).

**Figure 1:**
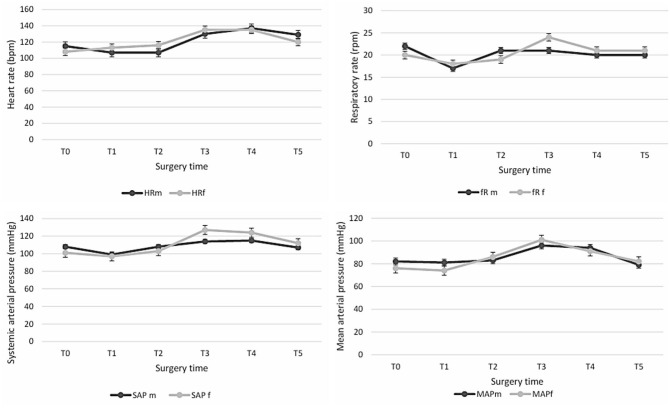
Comparison between groups at the six surgical time points (T0, T1, T2, T3, T4 and T5). Median HR, fR, SAP and MAP values in the OVE*m* and OVE*f* groups. fR = respiratory rate; HR = heart rate; MAP = mean arterial pressure; OVE*f* = flank ovariectomy; OVE*m* = midline ovariectomy; SAP = systolic arterial pressure

A comparison between the two groups showed no significant differences in the haemodynamic variables over the six surgical time points (*P* >0.05) (Figure 1).

In total, 18 analgesic rescues were performed in the OVE*m* group (T1, n = 1; T2, n = 3; T3, n = 8; T4, n = 5; T5, n = 1) and 13 were performed in the OVE*f* group (T3, n = 7; T4, n = 6); however, no significant differences were observed between the groups (*P* = 0.123).

## Discussion

This study suggests that OVE*f* is a faster technique and is associated with smaller surgical incisions than OVE*m*; however, there are no differences between OVE*m* and OVE*f* regarding intraoperative nociception, leading us to believe that both surgical techniques can be recommended for feral or stray cat sterilisation programmes.

As it is impossible or difficult to assess postoperative pain and administer postoperative analgesia, and there are no validated pain scales for feral or stray cats, our results support the idea that both techniques may be associated with similar levels of pain. Given the importance of intraoperative nociception for the postoperative period and the development of chronic or neuropathic pain,^13,14^ we believe that both techniques may produce similar degrees of postoperative pain in this type of feline population, as found in a study carried out on indoor cats.^8^

Although the cats in this study were randomised to the two surgical approaches, no significant differences in body weight and BCS were observed. In feral or stray cats, these two variables do not appear to be very important in the choice of surgical approach, as these cat populations generally have a low weight and BCS. However, in lean cats, such as those in these populations, lateral recumbency for the flank approach is easier to perform than dorsal recumbency for the ventral midline approach. A high weight and BCS may become important factors in the choice of approach to be used, as the OVE*f* approach may make it more difficult to access the ovary contralateral to the abdominal incision, making this approach contraindicated in obese cats.^10^

Significant differences in incision size were observed between the OVE*m* and OVE*f* approaches. Our results are in line with those of other studies,^7,16^ as the incision size was smaller in the OVE*f* approach. A possible explanation for this may be related to the ease of locating the reproductive organs through this approach.

Contrary to other studies,^8^ we did not standardise the incision size as this would not correspond to a real clinical context in stray or feral cat sterilisation programmes as this factor depends on the criteria and experience of the surgeon. Incision size and location are important in feral or stray cats as these animals are quickly released after sterilisation. The OVE*f* approach is associated with a smaller incision in the flank, and the absence of the weight of the abdominal organs on the lateral abdominal wall reduces the risk of wound dehiscence and evisceration and facilitates postoperative control from a distance when compared with the OVE*m* approach.^10,17^ OVE*f* can be performed via the right or left lateral flank approach.^10^ In our study, we used the left flank approach as this was the surgeon’s preference.

Mean surgery times were significantly higher for the OVE*m* approach. Compared with other studies,^11^ our surgery times were much higher in both approaches; however, this difference is related to the collection of intraoperative data as we waited for the maximum values of the haemodynamic parameters after each surgical stimulus before collecting data. As in other studies,^7,11^ the OVE*f* technique was associated with shorter surgery times, which, although not a critical point, may contribute to more surgeries being performed in a shorter time in sterilisation programmes where a large number of cats are neutered.

We believe that faster access to and closure of the abdominal cavity as well as faster identification of the reproductive structures may be reasons for the lower mean surgery times with the OVE*f* approach. Although the operations were performed by two surgeons, both equally familiar with the two approaches, this should be considered a limitation of our study as it may have biased our results.

Existing studies on this topic only address postoperative pain,^8^ and there are currently no studies evaluating intraoperative nociception while using both techniques, despite its importance for surgical recovery. Although no significant differences in nociception were observed between the two techniques, the intra-group analysis revealed that in the OVE*f* approach, the T3 and T4 surgical time points (removal of ovaries) were associated with a significant increase in HR and SAP. This suggests that the exteriorisation and removal of the ovarian pedicle in this approach may cause more intraoperative nociception than the same step during OVE*m*, particularly with the contralateral ovarian pedicle, which is in line with the veterinary literature,^10^ as greater stretching is required to exteriorise this pedicle.

This assumption is supported by the fact that the 13 analgesic rescues in the OVE*f* approach occurred at surgery time points T3 (n = 7) and T4 (n = 6) (removal of ovaries), likely a technique with more visceral pain,^11^ although no significant differences were observed between the two approaches.

Despite no significant differences observed between the surgical approaches, analysis of the analgesic rescue performed in the OVE*m* approach shows a different pattern from the OVE*f* approach, with rescue occurring during the application of surgical clamps (T1, n = 1), at the skin incision (T2, n = 3), during removal of the ovaries (T3, n = 8; T4, n = 5) and during the abdominal wall closure (n = 1), which contradicts a statement in another study^11^ that the sensitivity of the flank skin and musculature is greater than that of the midline skin and linea alba musculature.

Laparoscopic-assisted OVE is considered the technique of choice for feline OVE;^7^ however, the economic costs and technical aspects associated with this technique make its use in feral and stray cat sterilisation programmes unfeasible.

The limitations of this study relate to the lack of postoperative data collection, as the feral behaviour of these cats prevents this from being measured, and these animals are monitored remotely postoperatively. These data would include pain assessment, surgical wound inspection and surgical wound complications. There are currently no validated pain scales for feral or stray cats;^18^ however, it would be important to see if there is a correlation between intraoperative nociception and postoperative pain. Other limitations include the lack of invasive blood pressure monitoring, which precluded continuous and real-time assessment of the haemodynamic effects of surgery, and the lack of objective measurement of end-tidal isoflurane. Preventive analgesia with methadone and dexmedetomidine interfered with the nociceptive pattern in both approaches; however, animal welfare and pain control are fundamental.

## Conclusions

Our findings suggest that there is no strong reason based on intraoperative nociception to advocate a preferred surgical approach for feline OVE in sterilisation programmes; however, based on the duration of surgery and incision size, the OVE*f* approach could increase the number of animals sterilised and reduce the risk of dehiscence by allowing smaller incisions in animals where postoperative wound control is difficult or impossible.
